# C/EBPβ regulates the JAK/STAT signaling pathway in triple‐negative breast cancer

**DOI:** 10.1002/2211-5463.13138

**Published:** 2021-03-17

**Authors:** Shu Wang, Dian Xia, Xianzhi Wang, Haowei Cao, Chaoshen Wu, Zhaoran Sun, Daoyong Zhang, Hao Liu

**Affiliations:** ^1^ Jiangsu Key Laboratory of Brain Disease and Bioinformation Xuzhou Medical University China; ^2^ School of Pharmacy Bengbu Medical College China

**Keywords:** C/EBPβ, JAK, STAT, Triple‐Negative Breast Cancer

## Abstract

C/EBPβ is a member of the CCAAT/enhancer‐binding protein (C/EBP) family, which consists of a number of b‐ZIP transcription factors. Although C/EBPβ has been implicated in the development of certain cancers, including breast cancer, it remains unknown whether dysregulation of C/EBPβ in breast cancer is subtype‐specific. Moreover, the underlying mechanisms by which C/EBPβ regulates breast cancer carcinogenesis are not fully understood. Here, we present evidence that C/EBPβ is specifically overexpressed in human TNBC samples, but not in non‐TNBC samples. C/EBPβ depletion dramatically suppressed TNBC cell growth, migration, invasion, and colony formation ability. A subsequent mechanistic study revealed that the JAK/STAT signaling pathway was upregulated in C/EBPβ_high TNBC samples compared with C/EBPβ_low TNBC samples. C/EBPβ ChIP‐seq and qPCR were performed to demonstrate that C/EBPβ directly binds to and regulates JAK/STAT signaling pathway genes in TNBC. Taken together, our data indicate the oncogenic role of C/EBPβ in human TNBC and reveal a novel mechanism by which C/EBPβ promotes TNBC carcinogenesis.

## Graphical abstract

C/EBPβ is a b‐ZIP transcription factor. Here, we present evidence that C/EBPβ is specifically overexpressed in human TNBC samples, but not in non‐TNBC samples. C/EBPβ depletion dramatically suppresses TNBC cell growth, migration, invasion, and colony formation ability. Subsequent mechanistic studies reveal that C/EBPβ directly binds to and regulates JAK/STAT signaling pathway genes in TNBC.

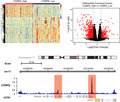

**Abbreviations**
C/EBPβ: CCAAT/enhancer‐binding protein beta
TNBC: triple‐negative breast cancer

C/EBPβ is a member of the CCAAT/enhancer‐binding protein (C/EBP) family, whose members share a highly conserved leucine‐zipper dimerization domain at the C‐terminal and an adjacent basic DNA‐binding region. The less conserved N‐terminal activation domain interacts with various transcriptional coactivators and general transcriptional factors [1, 2, 3, 4]. C/EBPs act as dimers to bind to DNA in a sequence‐specific manner to regulate transcription of the target genes [5, 6, 7]. Increasing evidence shows that C/EBPs play important roles in cell growth, differentiation, apoptosis, inflammation, aging, and neuronal autophagy [8, 9, 10, 11, 12, 13].

Emerging evidence has also implicated the crucial regulatory roles of C/EBPβ in a few types of cancer, including breast cancer, pancreatic cancer, sarcoma, and liver cancer [14, 15, 16, 17]. Although C/EBPβ is required for normal mammary gland growth and differentiation [18], its overexpression is capable of transforming normal human mammary epithelial cells, as evidenced by gaining anchorage independence, epithelial‐to‐mesenchymal transition marker gene expression, and the acquisition of an invasive phenotype [17]. These events are evidence that precisely regulated C/EBPβ expression is critical in normal mammary gland development, and dysregulation leads to breast carcinogenesis. Moreover, C/EBPβ is shown to be essential in maintaining mammary stem cells and luminal cell lineage commitment [19]. Although studies have indicated that C/EBPβ has important roles in a few types of cancer, the regulatory role of C/EBPβ has not been specifically and thoroughly studied in triple‐negative breast cancer (TNBC), and the underlying mechanisms remain largely unknown.

In the present study, we attempted to dissect the potential role of C/EBPβ specifically in TNBC and to understand the underlying mechanisms.

## Materials and methods

### Data source

mRNA expression data and clinical phenotype data of breast cancer from The Cancer Genome Atlas (TCGA) were downloaded from the UCSC Xena TCGA Hub. This included 1097 primary breast cancer samples and 113 normal breast tissue samples. According to the clinical phenotypes, 122 cases were TNBC and 617 cases were non‐TNBC, whereas the remaining cases had at least one of the progesterone receptor (PR) status, ER status, and HER‐2 status missing.

### Cell culture and RNAi

The TNBC cell line BT549 was originally purchased from ATCC. BT549 cells were cultured in RPMI 1640 medium (Invitrogen, Invitrogen, CA, USA) supplemented with 10% FBS. The shRNAs targeting C/EBPβ and the control scrambled sequence were as follows: C/EBPβ‐sh1, GCCGCCGCCTGCCTTTAAATC; C/EBPβ‐sh2, GCCCTGAGTAATCGCTTAAAG; shControl, TTCTCCGAACGTGTCACGT. Lentivirus preparation was performed as previously described [20]. The lentivirus was then used to infect BT549 cells to knock down C/EBPβ expression followed by selection using puromycin.

### Antibodies

The antibodies used in this study include anti‐C/EBPβ (Cat. No. sc‐7962; SANTA CRUZ, Santa Cruz, CA, USA) and anti‐STAT5A (Cat. No. 13179‐1‐AP; ProteinTech, ProteinTech, IL, USA).

### Quantitative real‐time PCR

RNA extraction, reverse transcription, and quantitative real‐time PCR were carried out as previously described [20].

### EdU incorporation assay and clonogenicity formation assay

BT549 cells were seeded at a density of 3 × 10^3^ cells per well in 96‐well plates and were incubated overnight. The media was prepared using EdU (5‐ethynyl‐2’‐deoxyuridine) for a final concentration of 50 μm, and the plates were incubated in a cell culture incubator for 2 h. The EdU incorporation assay was then performed using an EdU *In vitro* Imaging Kit (Ribo Bio, Guangdong, China) following the manufacturer’s protocol. The tumor cell colony‐forming ability was evaluated as previously described [20].

### Wound‐healing assay and Transwell cell invasion assay

Wound‐healing and Transwell cell invasion assays were performed as previously prescribed [20].

### Gene Set Enrichment Analysis

C/EBPβ expression in TNBC was divided into C/EBPβ_high and C/EBPβ_low expression groups using the median value. For the Gene Set Enrichment Analysis (GSEA), upregulated genes in the C/EBPβ_high group were used with the clusterProfiler R package.

## Results

### C/EBPβ expression was dysregulated in human triple‐negative breast cancer

**Fig. 1:**
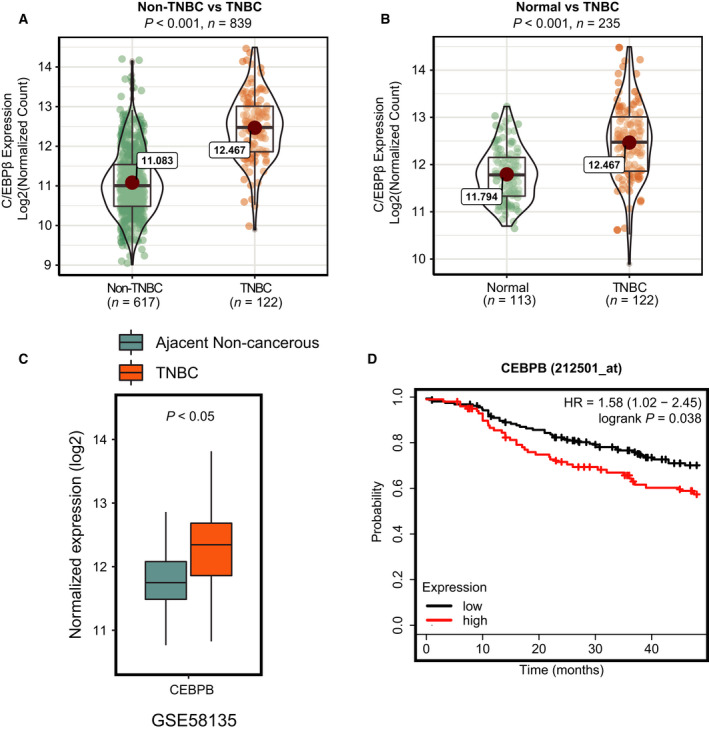
C/EBPβ is overexpressed in human TNBC. (A, B) The mRNA expression data obtained from the TCGA project were used to examine C/EBPβ expression in human TNBC (*n* = 122), non‐TNBC (*n* = 617), and normal breast tissue (*n* = 113) samples. The phenotype data obtained from the UCSC xena TCGA data hub were used to define TNBC and non‐TNBC samples. (C) RNA‐seq data from GSE58135 was used to assess C/EBPβ expression in human TNBC. (D) The Kaplan–Meier plotter (http://kmplot.com/analysis/) survival analysis for the relationship between the survival time of TNBC patients and C/EBPβ expression.

The study was designed to investigate the potential regulatory role of C/EBPβ in TNBC. To this end, we first compared C/EBPβ expression levels in TNBC samples (*n* = 122) versus non‐TNBC samples (*n* = 617) using data obtained from the UCSC Xena data hub. We found that the C/EBPβ was aberrantly overexpressed (more than twofold) in TNBC compared with non‐TNBC samples (Fig. 1A). Moreover, the C/EBPβ mRNA level in TNBC was also significantly higher than that in normal breast tissue samples (Fig. 1B). We also assessed the C/EBPβ expression in TNBC using another dataset (GSE58135) and observed a similar overexpression pattern in TNBC samples compared with the noncancerous adjacent samples (Fig. 1C). More importantly, we also observed a significant association between C/EBPβ expression and poor survival probability among TNBC patients, indicating the promising prognostic value of C/EBPβ in TNBC (Fig. 1D). Taken together, these results indicate that C/EBPβ may play a promoting role specifically in TNBC.

### C/EBPβ regulates TNBC cell proliferation, colony formation, migration, and invasion

**Fig. 2:**
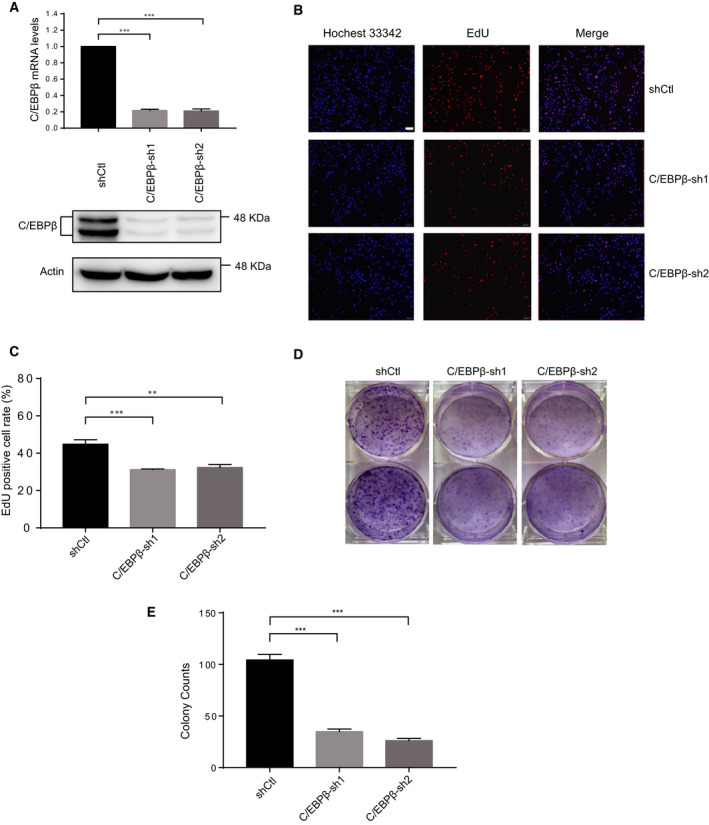
Knockdown of C/EBPβ inhibits TNBC cell proliferation and colony‐forming ability. (A) The knockdown efficiency of C/EBPβ was verified at the levels of transcription and translation. (B, C) The EdU proliferation assay was performed to determine the effect of C/EBPβ knockdown on TNBC cell proliferation. Three independent experiments were carried out, and the results were represented as mean + SD Scale bar, 100 μm. (D, E) The clonogenicity formation assay was performed to assess the effect of C/EBPβ knockdown on the colony‐forming ability of TNBC cells. Three independent experiments were carried out, and the results were represented as mean + SD. **P < 0.01, ***P < 0.001, unpaired *t*‐test.

To directly assess the regulatory role of C/EBPβ in TNBC, we knocked down C/EBPβ expression in BT549 cells. Knockdown efficiency was evaluated by real‐time quantitative PCR and western blotting (Fig. 2A). On the other hand, the EdU proliferation assay was used to assess the regulatory role of C/EBPβ in TNBC cell proliferation. As shown in Fig. 2B,C, C/EBPβ knockdown significantly inhibited TNBC cell proliferation. Next, we assessed the effect of C/EBPβ knockdown on TNBC cell colony formation using a clonogenic assay. The results showed that C/EBPβ knockdown dramatically impaired the colony formation ability of TNBC cells (Fig. 2D,E).

**Fig. 3:**
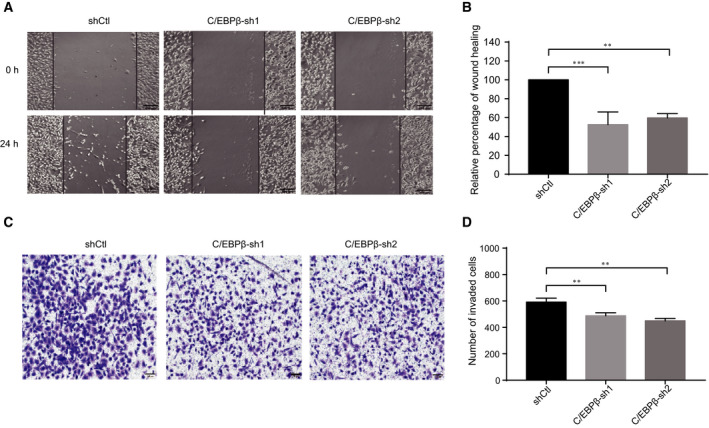
Knockdown of C/EBPβ inhibits TNBC cell migration and invasion. (A, B) The wound‐healing assay was carried out to examine the effect of C/EBPβ knockdown on TNBC cell migration. Three independent experiments were carried out, and the results were represented as mean + SD. Scale bar, 200 μm. (C, D) The Transwell invasion assay was performed to examine the effect of C/EBPβ knockdown on TNBC cell invasion. Three independent experiments were carried out, and the results were represented as mean + SD. Scale bar, 100 μm. **P < 0.01, ***P < 0.001, unpaired *t*‐test.

To further elucidate the role of C/EBPβ in TNBC, we also investigated the regulatory role of C/EBPβ in TNBC cell migration and invasion, which are both important processes in breast cancer metastasis. To this end, wound‐healing and Transwell cell invasion assays were performed. As shown in Fig. 3A,B, we observed slower migration of TNBC cells upon C/EBPβ knockdown. Furthermore, the Transwell cell invasion assay results demonstrated that C/EBPβ knockdown dramatically impaired the invasiveness of TNBC cells (Fig. 3C,D). These results together support the conclusion that C/EBPβ plays a role in promoting TNBC.

### C/EBPβ regulates JAK/STAT signaling pathway genes in TNBC

**Fig. 4:**
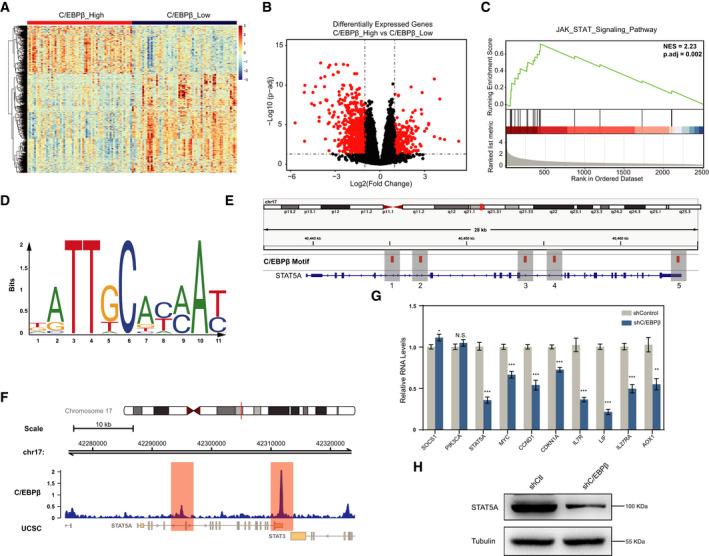
C/EBPβ regulates the JAK/STAT signaling pathway in human TNBC. (A) Hierarchical cluster analysis of DEGs (|logFC| > 0.5 & *P* < 1E‐4) between C/EBPβ_high and C/EBPβ_low TNBC samples. Each cell in the matrix represents the expression of a gene in an individual sample. (B) Volcano plot of differential gene expression between C/EBPβ_high and C/EBPβ_low TNBC samples. Each dot represents one gene that had detectable expression in the RNA‐seq experiment. The horizontal dashed line marks the threshold (*P*.adj < 0.05) for defining a gene as significantly upregulated or downregulated in C/EBPβ_high samples compared with C/EBPβ_low samples. The vertical dashed lines represent twofold differences in expression. Significantly DEGs (|log_2_(FC)| > 1 and *P*.adj < 0.05) are marked in red. (C) Gene set enrichment analysis (GSEA) score curves. The JAK/STAT signaling pathway enrichment plot is shown. Black bars represent the position of genes of the JAK/STAT signaling pathway in the ranked list together with the running enrichment score (plotted in green). (D) The C/EBPβ binding motif was obtained from the JASPAR database. The character and size of each logo represent the proportion of a base at a specific site. (E) The positions of potential C/EBPβ‐binding sites at the STAT5A gene, defined by its binding motif, are shown. (F) C/EBPβ ChIP‐seq data (GSM2330565) were analyzed to determine C/EBPβ‐binding signals at the STAT5A gene. (G) qRT‐PCR assays were performed to examine the effect of C/EBPβ knockdown on JAK/STAT signaling pathway genes. Three independent experiments were carried out, and the results were represented as mean + S.D. *P < 0.05, **P < 0.01, ***P < 0.001. Unpaired *t*‐test. (H) Western blotting assay was carried out to confirm the change in STAT5A expression at the protein level upon C/EBPβ knockdown.

We next explored the underlying mechanisms by which C/EBPβ promotes TNBC carcinogenesis. To this end, we divided the TNBC cases into two groups, the C/EBPβ_high group and the C/EBPβ_low group, based on the median C/EBPβ expression level in TNBC. Then, we compared the two groups and identified the differentially expressed genes (DEGs) using the DEseq2 R package. The gene expression patterns between the C/EBPβ_high group and the C/EBPβ_low group were evidently distinct (Fig. 4A,B). The upregulated genes in the C/EBPβ_high group were then used in the GSEA analysis, and the results indicate that the JAK/STAT signaling pathway genes were enriched and upregulated in the C/EBPβ_high group compared with the C/EBPβ_low group (Fig. 4C).

As a transcriptional factor, C/EBPβ directly binds to DNA in a sequence‐specific manner to regulate the expression of target genes. We were interested in determining whether these JAK/STAT signaling pathway genes were directly bound by C/EBPβ at the chromatin level. Since C/EBPβ recognizes and binds to specific DNA sequences, we first tried to identify potential C/EBPβ‐binding sites at the JAK/STAT signaling pathway genes. We used the STAT5A gene as an example in this study. We first obtained a C/EBPβ‐binding DNA sequence motif from the JASPAR database (Fig. 4D) and then loaded the bed file to the IGV software to identify C/EBPβ‐binding motif sites at the STAT5A gene locus. As seen in Fig. 4E, we identified five potential C/EBPβ‐binding sites throughout the STAT5A gene body region.

However, the existence of a C/EBPβ‐binding motif sequence does not necessarily mean that C/EBPβ is actually bound. The binding of C/EBPβ to the motif sequence is regulated by many other factors, and its genome‐binding profile varies from cell to cell. To verify whether these sites are actually bound by C/EBPβ in TNBC, we analyzed the ChIP‐seq data for C/EBPβ binding in SUM159PT cells, a TNBC cell line. The results showed that there was a strong C/EBPβ‐binding signal at the 3′ end of the STAT5A gene body region, corresponding to the 5^th^ C/EBPβ‐binding motif locus identified above (Fig. 4F). Weak binding of C/EBPβ was also observed in the middle region, corresponding to the 2nd C/EBPβ‐binding motif locus. To further demonstrate the regulatory role of C/EBPβ in TNBC cells, we assessed the effect of C/EBPβ depletion on the expression of genes involved in the JAK/STAT pathway in TNBC cells. The results demonstrated that the majority of JAK/STAT signaling pathway genes examined here were downregulated upon C/EBPβ knockdown, including STAT5A (Fig. 4G,H), suggesting a positive regulatory role of C/EBPβ in the expression of these genes. Taken together, our results support that C/EBPβ directly binds to JAK/STAT signaling pathway genes and regulates their expression in TNBC.

## Discussion

TNBC is an aggressive subtype of breast cancer in which the expression of estrogen receptor (ER), PR, and human epidermal growth factor receptor‐2 is absent [21, 22]. The survival time of TNBC patients is shorter, and the mortality rate is higher compared with that of non‐TNBC patients. Moreover, TNBC is highly invasive, and nearly half of TNBC patients will have distant metastasis. Here, we examined the regulatory role of C/EBPβ, specifically in TNBC. Our data showed that C/EBPβ is significantly upregulated in TNBC samples compared with non‐TNBC and normal breast tissue samples. C/EBPβ depletion in TNBC cells inhibited their proliferation rate and colony formation ability. Since TNBC is a highly invasive breast cancer subtype, we also investigated the role of C/EBPβ in TNBC cell migration and invasion, which are both critical processes in cancer metastasis. Our data demonstrated that C/EBPβ knockdown dramatically inhibited TNBC cell migration and invasion. A subsequent mechanistic study showed that C/EBPβ binds to JAK/STAT signaling pathway genes and regulates their expression. Given that the dysregulation of the JAK/STAT signaling pathway plays an instrumental role in breast cancer development and metastasis as well as in other types of cancer [23, 24, 25, 26, 27, 28, 29], we concluded that C/EBPβ promotes TNBC carcinogenesis probably via the regulation of the JAK/STAT signaling pathway.

C/EBPβ is an intronless gene, and due to multiple translation start sites within the mRNA, three distinct isoforms of C/EBPβ proteins (LAP*, LAP, and LIP) are expressed. The C/EBPβ isoform LAP has been previously reported to have an impact on the tumor microenvironment in TNBC [30]. The involvement of LAP in tumor immunity indicates that C/EBPβ plays a much broader role in TNBC than we previously thought. Although we observed C/EBPβ overexpression in TNBC samples, we do not know which specific isoform is eventually overexpressed at the protein level. Previous studies showed that LAP* was the only C/EBPβ protein expressed in normal mammary tissue and was absent from breast cancer cells by contrast. Instead, the isoform LAP was reportedly upregulated in primary breast tumors [17]. Therefore, we hypothesize that LAP is the isoform that is overexpressed in TNBC as a result of upregulated C/EBPβ mRNA levels. However, further experimental data are needed to confirm this hypothesis.

## Conclusion

In the present study, we observed elevated C/EBPβ expression specifically in TNBC samples compared with non‐TNBC and normal breast tissue samples. Further studies showed that C/EBPβ knockdown inhibited TNBC carcinogenesis. Moreover, a mechanistic study revealed that C/EBPβ binds to and regulates JAK/STAT signaling pathway genes in TNBC cells, indicating that C/EBPβ may promote TNBC carcinogenesis by regulating the JAK/STAT signaling pathway. In conclusion, our results demonstrate a promoting role of C/EBPβ in human TNBC and reveal a novel underlying mechanism.

## Conflict of interest

The authors declare no conflict of interest.

## Author contributions

SW, DX, XW, HC, CW, and ZS performed experimental work; SW, DX, XW, HC, DZ, and HL analyzed experimental data; DZ performed bioinformatic analyses; DZ and HL designed and supervised the study; DZ and HL wrote the manuscript. All authors read and approved the final manuscript.

## Data Availability

The data will be available from the corresponding author upon reasonable request.
